# A145 EFFICACY AND SAFETY OF UPADACITINIB MAINTENANCE THERAPY IN PATIENTS WITH MODERATELY TO SEVERELY ACTIVE ULCERATIVE COLITIS: RESULTS FROM A RANDOMIZED PHASE 3 STUDY

**DOI:** 10.1093/jcag/gwab049.144

**Published:** 2022-02-21

**Authors:** R Panaccione, S Danese, W Zhouwen, A Pangan, X Hébuterne, H Nakase, G D’Haens, J Panes, J Lindsay, P Higgins, E Loftus, W Sandborn, W Xie, Y Sanchez gonzalez, J Liu, M Weinreich, S Vermeire

**Affiliations:** 1 University of Calgary, Calgary, AB, Canada; 2 IRCCS Ospedale San Raffaele, Milano, Lombardia, Italy; 3 AbbVie Inc, North Chicago, IL; 4 Universite Cote d’Azur, Nice, Provence-Alpes-Cote d’Azu, France; 5 Sapporo Ika Daigaku Igakubu Daigakuin Igaku Kenkyuka, Sapporo, Hokkaido, Japan; 6 Barts Health NHS Trust, London, London, United Kingdom; 7 University of Michigan Michigan Medicine, Ann Arbor, MI; 8 Mayo Clinic Minnesota, Rochester, MN; 9 University of California San Diego, La Jolla, CA; 10 Universiteit van Amsterdam, Amsterdam, Noord-Holland, Netherlands; 11 Hospital Clinic de Barcelona, Barcelona, Catalunya, Spain; 12 Katholieke Universiteit Leuven Universitaire Ziekenhuizen Leuven Campus Gasthuisberg, Leuven, Flanders, Belgium

## Abstract

**Background:**

Upadacitinib (UPA), an oral selective and reversible JAK inhibitor, demonstrated significantly greater efficacy compared with placebo (PBO) for induction of remission in patients with moderately to severely active ulcerative colitis (UC) in two phase 3 induction trials.

**Aims:**

Evaluate safety and efficacy of 52 weeks of UPA 15 mg QD (UPA15) and 30mg QD (UPA30) compared to placebo in patients achieving clinical response following UPA 45 mg treatment in the induction trials.

**Methods:**

The primary analysis (n=451) evaluated efficacy and safety of UPA15 and UPA30 compared to PBO as maintenance therapy. The primary endpoint was clinical remission via adapted Mayo score at wk 52. Ranked secondary endpoints included endoscopic improvement, maintenance of clinical remission, corticosteroid-free clinical remission, maintenance of endoscopic improvement, endoscopic remission, maintenance of clinical response and Histologic-endoscopic mucosal improvement (HEMI).

**Results:**

Baseline characteristics were similar between all treatment groups. Both UPA15 and UPA30 met the primary endpoint, and all secondary endpoints. Significantly greater percentages of patients receiving UPA15 and UPA30 vs. PBO achieved clinical remission (42.3% and 51.7%, vs. 12.1%), endoscopic improvement (48.7% and 61.6%, vs. 14.5%), maintenance of clinical remission (59.2% and 69.7%, vs. 22.2%), corticosteroid-free clinical remission (57.1% and 68.0%, vs. 22.2%), maintenance of endoscopic improvement (61.6% and 69.5%, vs. 18.9%), endoscopic remission, (24.2% and 25.9%, vs. 5.6%) and HEMI (34.8% and 49.3%, vs. 11.8%) (p<0.001 for all endpoints). UPA15 and UPA30 were both well-tolerated and no new safety signals were observed. Rates for serious adverse events (AEs) and AEs leading to treatment discontinuation were similar between UPA15 and UPA30 groups and lower compared to the PBO group. Most common AEs were nasopharyngitis and creatine phosphokinase elevation among UPA groups and UC exacerbation within the PBO group (30.2%). Herpes zoster was only reported in UPA groups (3.9%-4.1%). Similar rates of malignancy excluding NMSC were seen within all groups (0.7%-1.3%). MACE were only reported among patients receiving PBO (0.7%), while VTE were only found with UPA30 (1.3%).

**Conclusions:**

In patients responding to UPA induction therapy, both UPA15 and UPA30 were safe and effective as maintenance treatment at 52 wk for all primary and secondary endpoints. Patients receiving UPA30 responded approximately 10% better for most endpoints compared to those receiving UPA15. Both doses were well-tolerated, with no new safety signals observed.

**Funding Agencies:**

AbbVie

